# Clinical and Technical Considerations of Luting Agents for Fixed Prosthodontics

**DOI:** 10.1155/2012/565303

**Published:** 2012-06-25

**Authors:** Cornelis H. Pameijer, Per-Olof Glantz, Anthony von Fraunhofer

**Affiliations:** ^1^Department of Reconstructive Sciences, School of Dental Medicine, University of Connecticut, 263 Farmington Avenue, Farmington, CT 06030, USA; ^2^Faculty of Odontology, Malmö University, 20506 Malmö, Sweden; ^3^School of Dentistry, University of Maryland, Baltimore, MD 21201, USA

The selection of an appropriate luting agent for final cementation of fixed restorations is no longer an easy decision. The numerous materials from which restorations can be fabricated demand a thorough knowledge of not only the material(s) they are made of but also knowledge of the properties of luting agents. No longer can zinc phosphate cement be used for all fixed restorations that need cementation, as has been the case since its inception, late 1800s, until the 1970s. With the introduction of new restorative materials and clinical applications such as nonprecious alloys, feldspathic veneers, all-ceramic restorations, zirconium, lithium disilicate, the demand for more suitable cements developed somewhat in tandem with new materials and clinical techniques. As a result, today's market is flooded with cements, and more are being introduced on a regular basis. Even for the expert and researcher, the combination of these cements with the large variety of restorative materials would be a daunting task to test, and frequently the clinician has to hope that the choice is the proper one. 

This special issue presents a review of luting agents and attempts to bring the practitioner up to date with new developments. Of particular promise in restorative dentistry are the bioactive luting agents that have sealing and remineralizing potential. In this regard, a common misconception is that a luting agent must have high retentive values of its own. The reality is that a luting agent in addition to being an adhesive primarily acts as a seal or gasket to prevent bacteria from penetrating the tooth-restoration interface. Retention is primarily determined by the mechanical resistance and geometry of the preparation. 

The authors would like to thank the many reviewers who participated in the evaluation of the twenty submitted manuscripts. Of these, eight manuscripts were accepted and are presented here.

A comprehensive literature review by C. Pameijer highlights composition, physical properties, and clinical applications of luting agents since their inception late 1880s until today with an introduction of the latest developments. A chart with clinical indications is included, which is intended as a guide to selecting an appropriate luting agent. It should be noted that the author pointed out that this chart is strictly based on personal experience and research; much of which has been conducted by the author. 

T. Abo and coworkers compared the microtensile bond strengths (*μ*TBSs) of three self-adhesive luting cements and a control (Panavia F 2.0) between ceramics and resin cores and examined their relation to cement thickness of 25, 50, 100, or 200 *μ*m. The three self-adhesive cements scored lower *μ*TBSs than the control that required etching and a primer before cementation. A cement thickness of 50 or 100 *μ*m tended to induce the highest *μ*TBS, which are clinical parameters that are usually accepted in spite of the recommended 40 *μ*m that is advocated by the American Dental Association.

I. Costa Sousa Oliveira and coworkers assessed the effect post-cementation waiting time for core preparation of cemented cast posts and cores had on retention in the root canal. They tested two different luting materials on sixty extracted human canines. The post and cores were fabricated in cast nickelchromium and luted with zinc phosphate (ZP) cement or resin cement. The specimens were divided into 3 groups (*n* = 10) according to the waiting time for core preparation: tested without preparation (control), after 15 minutes and after 1 week after the core cementation. A tensile load test was applied until failure. Significantly higher (*P* < 0.05) tensile strength values were obtained for ZP cement; however, core preparation and postcementation waiting time did not affect the retention strength. The authors concluded that ZP was the best material for intraradicular metal posts cementation. It should be noted, however, that the results do not necessarily apply to all metals that typically are used for post and core fabrication.

In an in vivo study by D. Gemalmaz and co-workers, disintegration of four luting agents was evaluated using a gold intraoral sample holder that had four holes of 1.4 mm diameter and 2 mm depth, which were filled with zinc phosphate (Phosphate Kulzer), a glass ionomer (Ketac Cem), a resin-modified-glass ionomer (Fuji Plus), and a resin cement (Calibra). The holder was soldered onto the buccal surface of an orthodontic band, which was cemented to the first upper molar in 12 patients. Impressions were made at baseline and 6, 12, and 18 months from which epoxy replicas were made, which were scanned with an optical scanner. Total volume loss was calculated. The rank order of mean volume loss was as follows: Phosphate cement > Ketac Cem = Fuji Plus = Calibra. Cement type and time had statistically significant effects on volume loss of cements (*P* < 0.001). Of interest here was the fact that the disintegration of glass ionomer and resin-based cements was not statistically significantly different. 

P. Kesrak and C. Leevailoj designed a study to determine the hardness of two light curing resin cements under five different ceramic discs measuring 0.5, 1.0, 1.5, and 2.0 mm in thickness. They concluded that resin cements polymerized under different ceramic materials and thickness showed statistically significant differences in Knoop hardness. 

A new hydraulic calcium silicate cement designed for restorative dentistry (Biodentine) was compared with a resin-modified glass ionomer cement (Ionolux) in open-sandwich restorations covered with a light-cured composite by S. Koubi and co-workers. Each group consisted of 5 samples including 5 for the positive and 5 for the negative control. After simultaneously thermocycling and mechanocycling using a fatigue cycling machine (1,440 cycles, 5–55°C; 86,400 cycles, 50 N/cm^2^), the specimens were then stored in phosphate-buffered saline to simulate aging for 1 year. The samples were then submitted to glucose diffusion. Statistical analysis of the data demonstrated that there was no difference between the Biodentine and the Ionolux group. The calcium silicate-based material performed as well as the resin-modified glass ionomer cement in open-sandwich restorations. 

Antibacterial properties are a desirable feature of luting agents. E. Unosson and co-workers designed a clinically relevant laboratory experiment and compared 4 cements (a self-etching cement, a glass ionomer cement, a hybrid calcium aluminate cement, and a zinc phosphate cement) to 2 reference cements (calcium aluminate and glass ionomer cement) and a negative control (PMMA). Antibacterial properties were tested after 10 minutes, 1 and 7 days using *Streptococcus mutans.* Viable bacteria in direct contact with the samples were determined with a metabolic assay containing resazurin. Furthermore, effect of F and pH was also evaluated. The results indicated that antibacterial properties in descending sequence were calcium aluminate cement > Ceramir Crown & Bridge > and RelyXTM Unicem. KetacTM Cem, Harvard Cement, and the reference glass ionomer cement showed bacteria contents either higher than or not significantly different from the PMMA control. Furthermore pH levels below 6.3 and above 9.0 were found to have negative effects on bacterial proliferation while the more basic the cement was, the stronger the antibacterial properties. These findings may explain tissue health around crown margins of certain cements that have a high pH. 

In the J. von Fraunhofer paper, an in-depth discussion on adhesion and cohesion is presented. The text is amply illustrated with detailed drawings that explain the basic principles in a graphic way. Adhesion principles in dentin bonding are also highlighted. It may seem to the reader that the basics that are discussed have no direct bearing on clinical dentistry. The opposite is true. Understanding these principles allows a practitioner to understand success and failure in dentistry much better and can contribute in the selection of an appropriate luting agent.

It is hoped that the information that is provided in this issue will help the practitioner in making prudent decisions that will offer patients a restoration that has a long life span and offers the dentist the assurance that his/her work will last for many years.

*Cornelis H. Pameijer* *Cornelis H. Pameijer* *Per-Olof Glantz* *Per-Olof Glantz* *Anthony von Fraunhofer* *Anthony von Fraunhofer*

